# Hepatitis E virus antibody prevalence in hunters from a district in Central Germany, 2013: a cross-sectional study providing evidence for the benefit of protective gloves during disembowelling of wild boars

**DOI:** 10.1186/s12879-015-1199-y

**Published:** 2015-10-22

**Authors:** A. Schielke, V. Ibrahim, I. Czogiel, M. Faber, C. Schrader, P. Dremsek, R. G. Ulrich, R. Johne

**Affiliations:** Robert Koch Institute (RKI), Berlin, Germany; Postgraduate Training for Applied Epidemiology (PAE, German Field Epidemiology Training Programme), Robert Koch Institute, Berlin, Germany; European Programme for Intervention Epidemiology Training (EPIET), European Centre for Disease Prevention and Control (ECDC), Stockholm, Sweden; Local Authority Wetteraukreis, Friedberg, Germany; Federal Institute for Risk Assessment (BfR), Berlin, Germany; Friedrich-Loeffler-Institut (FLI), Institute for Novel and Emerging Infectious Diseases, Greifswald-Insel Riems, Germany

**Keywords:** Hepatitis E virus, Seroprevalence, Wild boars, Hunting, Protective gloves

## Abstract

**Background:**

In Germany, 17 % of the general human population have antibodies to hepatitis E virus (HEV) (recomLine HEV-IgG/IgM immunoassay [Mikrogen GmbH]). Wild boars represent an animal reservoir for HEV genotype 3, which is the common genotype in Germany. We estimated the seroprevalence among hunters with contact to wild boars to identify factors that may be associated with past or present HEV infection.

**Methods:**

In 2013, the local veterinarian authority in a district in Central Germany attended meetings of hunters who provided blood specimens and completed a questionnaire collecting information on age, sex, hunting-related activities and consumption of wild boar meat. Specimens of wild boars were taken during drive hunts in this district during the season 2012/2013. All specimens were tested for HEV RNA and anti-HEV IgM and IgG antibodies. Log-binomial regression was used to estimate prevalence ratios (PR) for the hunters.

**Results:**

Of 126 hunters (median age 55; 94 % male) 21 % tested positive for anti-HEV IgG antibodies (95 % confidence interval [CI] 13–28 %) (recomWell HEV IgG assay [Mikrogen GmbH]). Anti-HEV prevalence was highest in the age group of the 70–79-year-olds (67 %; 95 % CI 39–95 %). Wild boars showed an average anti-HEV prevalence of 41 %. HEV RNA was detected in 4/22 (18 %) liver specimens and in 1/22 (4.5 %) muscle specimens. Most wild boars were tested positive for HEV RNA (3/10; 30 %) and HEV-specific antibodies (7/15; 47 %) in the southwestern part of the district. Hunters preferring this hunting ground had a lower anti-HEV prevalence when gloves were frequently used during disembowelling of wild boars compared to hunters using gloves never or infrequently (age-adjusted PR 0.12; 95 % CI 0.02–0.86).

**Conclusions:**

Hunters may benefit from wearing gloves when in contact with blood or body fluids of HEV animal reservoirs. Anti-HEV prevalence among the hunters of this study did not significantly differ from that of the general population suggesting that other factors play a major role in the epidemiology of HEV in Germany.

**Electronic supplementary material:**

The online version of this article (doi:10.1186/s12879-015-1199-y) contains supplementary material, which is available to authorized users.

## Background

Hepatitis E has been notifiable in Germany since 2001. Since then, the number of notified cases has been increasing steadily each year. Men between 50 and 69 years of age are the most affected group with 0.9 cases per 100,000 population [1]. Underreporting is expected due to asymptomatic and/or undiagnosed infections [2, 3].

The main route of transmission of HEV genotype (gt) 3, the common genotype in Europe, is zoonotic [4, 5]. A case-control-study conducted in Germany identified consumption of offal and wild boar meat as the main risk factors for an HEV infection [6]. Investigations of reservoir animals in Germany revealed a high proportion of domestic pigs and wild boars positive for anti-HEV antibodies or HEV RNA [7–11].

A seroprevalence study among healthy adults representative for the German general population revealed an anti-HEV prevalence of 17 % [3]. In Europe, anti-HEV prevalence in blood donors ranged between 0.23 % in Greece and 53 % in France [12, 13]. However, comparability of seroprevalence estimates is hampered by the use of different serological assays and the lack of a gold standard [14, 15].

In persons with occupational contact to pigs the anti-HEV prevalence is higher compared to the general population [14, 16, 17]. Especially slaughterers show higher anti-HEV prevalences compared to people without occupational exposure to pigs (42 *vs*. 16 %) [17]. In forest workers, anti-HEV prevalences of 18 % in Germany and 36 % in France were reported [16, 18].

During skinning and disemboweling of HEV animal reservoirs like wild boars and deer, hunters may have direct contact to blood or other body fluids in case they do not wear any barrier protection as for example protective gloves. In Japan, anti-HEV prevalence in wild boar hunters was significantly higher than in the general population (25 *vs*. 5.5 %) [19]. A seroprevalence study among healthy blood donors in France indicated an association between hunting and an increased prevalence of anti-HEV antibodies [20].

The objective of the here presented cross-sectional study was to estimate anti-HEV antibody prevalence among hunters with contact to wild boars and to identify factors that may be associated with past or present HEV infection.

## Methods

### Study design and data collection

**Fig. 1:**
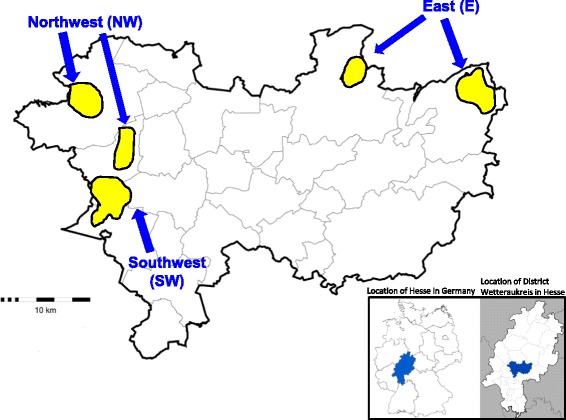
Map of the Wetteraukreis district, Hesse in Central Germany, 2013. Hunting grounds in this district are marked in yellow. These were grouped into three areas: East (E),Northwest  (NW) and Southwest (SW) (indicated by arrows). The location of Wetteraukreis district and Hesse in Central Germany are presented in the box

For this cross-sectional study, the local veterinarian authority recruited study participants during four meetings of hunters in the Wetteraukreis district in Hesse, Central Germany, at the beginning of 2013. After informed written consent of the participants, blood specimens were collected and a questionnaire was completed collecting information on age, sex as well as hunting ground, hunting activities and consumption of wild boar meat. Hunting grounds in the Wetteraukreis district were grouped into three areas: East (E), Northwest (NW) and Southwest (SW) (Fig. 1). Pivotal for the attribution of a hunter to a certain hunting ground was the location of the meeting he was attending assuming that this may reflect his or her preference for a certain region. In accordance with Article 25 paragraph 1 of the “German Infection Protection Act” a formal ethical review process and approval was not required. All study participants were informed of their results by the local authority. The data from the pseudonymized questionnaires were entered into an Excel data sheet and then imported into Stata 12 (Stata Corporation, College Station, TX; USA) for statistical analysis.

Additionally, specimens of wild boars (blood, muscle and liver tissue) were taken during different drive hunts in this district during the season 2012/2013. Human as well as wild boar specimens were tested for HEV-specific antibodies and HEV RNA as described below.

### Serological testing

For the serological testing of the human sera three Enzyme Linked Immunosorbent Assays (ELISAs) were used: (1) the recomWell HEV IgG assay, which is an indirect ELISA based on recombinant open reading frame (ORF) 2- and ORF3-derived antigens of gt1 and gt3 (Mikrogen GmbH, Neuried, Germany); (2) the recomWell HEV IgM assay (Mikrogen GmbH), which uses the same antigens as the IgG detecting version of the test; (3) the HEV Ab-ELISA kit (Axiom, Bürstadt, Germany), which is a double-antigen sandwich-ELISA based on the capsid protein of gt1. This test is species-independent and detects all classes of antibodies.

The porcine sera were tested using the HEV Ab-ELISA kit (Axiom). All tests were used according to their manuals provided by the manufacturers.

### Reverse transcription - polymerase chain reaction and phylogenetic analysis

Nucleic acids were isolated from serum specimens of hunters and wild boars using the NucliSENS® easyMag® device and reagents (bioMérieux Deutschland GmbH, Nürtingen, Germany) according to the manufacturer’s protocol. Specimens from liver and muscle tissue of wild boars were homogenized with mortar and pestle and subjected to RNA isolation with the RNeasy Mini Kit (Qiagen, Hilden, Germany). HEV-specific RNA was detected by real-time reverse - transcription polymerase chain reaction (RT-PCR) as previously described [21] using the Quantitect Probe RT-PCR Kit (Qiagen) in a 7500 Real-Time PCR System (Applied Biosystems, Foster City, CA, USA). Wild boar liver and muscle specimens tested positive by real-time RT-PCR were subjected to conventional RT-PCR as described by Herremans et al. [22] amplifying a 197 nucleotide (nt) fragment of ORF2. This RT-PCR was performed using the QIAGEN OneStep RT-PCR Kit (Qiagen) in a 2720 Thermal Cycler (Applied Biosystems). The amplification products were purified using the QIAquick DNA purification kit (Qiagen) and directly sequenced using the RT-PCR primers in an ABI 3730 DNA Analyzer (Applied Biosystems) [GenBank: KP127667 – KP127670]. Phylogenetic trees were constructed using a neighbour-joining method implemented in the MegAlign module of the DNASTAR software package (Lasergene, Madison, WI, USA) and bootstrap analysis was performed with 1000 trials and 111 random seeds.

### Statistical analysis

Among hunters, an acute HEV case was defined as a person who participated in one of the meetings in the Wetteraukreis district in 2013 and tested positive for HEV RNA and/or anti-HEV IgM. A subject tested positive only for anti-HEV IgG was defined as a case with a previous HEV infection. Accordingly, subjects tested negative for HEV RNA as well as anti-HEV IgM and IgG were defined as “non-cases”.

Subjects (a) less than 18 years old, (b) negating any hunting activity, (c) being a butcher or (d) for which the outcome result was missing were excluded from further analysis. The extent of contact between hunters and wild boars was categorized by the frequency of the use of protective gloves while skinning and/or disembowelling of wild boars. A new binary variable was created to differentiate between frequent (“always” and “nearly always”) or infrequent (“never”, “seldom” and “sometimes”) use of gloves.

Seroprevalences and 95 % confidence intervals (CI) were calculated using the results of both serological assays: recomWell IgG and Axiom, separately. Cohen’s kappa coefficient (κ) served as a measure of concordance between the two assays.

To compare the anti-HEV prevalences of the hunters from this study with that of the German general population, a large subsample (*n* = 4352) of sera originating from the 2008–2011 German Health Examination Survey for Adults (Deutscher Erwachsenen Gesundheitssurvey [DEGS]; www.degs-studie.de) was used to determine the baseline prevalence of anti-HEV antibodies in healthy adults in Germany [3]. The sera in DEGS were screened with the recomLine HEV-IgG/IgM immunoassay (Mikrogen GmbH). As the recomLine assay has the highest concordance with the recomWell IgG assay used in our study with κ = 0.80 [14] indicating a substantial concordance of the two assays [23], comparisons between the DEGS sera representing the German general population and the hunters from this study were based on these results. Pearson’s chi-square and Fisher’s exact statistics were used for significance testing. Results were considered as statistically significant if *p*-values were <0.05.

To identify factors that are associated with past or present HEV infection, prevalence ratios (PR) were estimated using univariable and stratified analysis as well as log-binomial regression.

For multivariable analysis, the results for the hunters above 70 years of age were excluded due to the possible presence of an unknown confounder and not being able to adjust for. Serology in the hunters (dichotomic: positive/negative) was defined as the dependent variable. In the final model, age group (in categories of 10 years), the extent of contact between hunters and wild boars and the hunting ground plus the according interaction term were included as independent variables.

## Results

### Seroprevalence in the hunters

In total, 137 persons with a median age of 54 (range 17–84 years of age) joining one of the four meetings for hunters in 2013 accepted to participate in this study; 93 % of them were males. Seven persons were working as butchers. Of these, 6 were positive for anti-HEV IgG in both assays. After application of the defined criteria, persons (a) younger than 18 years old (*n* = 1), (b) working as a butcher (*n* = 7), or (c) denying any hunting activities (*n* = 3) were excluded resulting in 126 subjects included in the further analysis as final sample. The median age was 55 years (range 22–84 years of age); 94 % were males. No HEV RNA was detected in the serum specimens of the hunters by real-time RT-PCR. Only one of the hunters was positive for HEV-specific IgM. The apparent anti-HEV IgG prevalence in the hunters was 21 % (95 % CI 13–28 %) or 38 % (95 % CI 29–47 %) based on the results of the recomWell IgG or Axiom assay, respectively, with κ = 0.56 suggesting a moderate concordance according to criteria by Landis & Koch [23]. There was no difference in the seroprevalence between men and women regardless of the used assay. Likewise, there was no substantial difference in the seroprevalence of the three hunting grounds. By dividing the sample into seven age groups, seroprevalence was found to increase with age peaking in the 70–79 years-olds based on the results of the Axiom assay. This general trend was confirmed by the results of the recomWell IgG assay, where the seroprevalence in the 70–79 years-olds was considerably higher compared to the other age groups (Table 1).

**Table 1:** Anti-HEV prevalences for hunters, Wetteraukreis district, Hesse in Central Germany, 2013

		recomWell IgG assay		Axiom assay	
		Seroprevalence	95 % CI	Seroprevalence	95 % CI
Age groups	20–29	0 % (0/7)		14 % (1/7)	0-43*
	30–39	0 % (0/9)		0 % (0/9)	
	40–49	16 % (4/25)	1.1–31	24 % (6/25)	7–41
	50–59	20 % (7/35)	6.4–34	43 % (15/35)	26–60
	60–69	19 % (7/36)	3.2–33	47 % (17/36)	31–64
	70–79	67 % (8/12)	39–95	67 % (8/12)	39–95
	80–89	0 % (0/2)		50 % (1/2)	0–100*
	Total	21 % (26/126)	13–28	38 % (48/126)	29–47
Sex	male	20 % (24/118)	13–28	39 % (46/118)	30–48
	female	25 % (2/8)	0–57*	25 % (2/8)	0–57*
Hunting area	East (E)	26 % (9/34)	11–42	52 % (17/34)	34–69
	Northwest (NW)	14 % (9/65)	5–22	31 % (20/65)	19–42
	Southwest (SW)	30 % (8/27)	12–47	41 % (11/27)	22–60

### Comparison of seroprevalence between hunters and general population

**Fig. 2:**
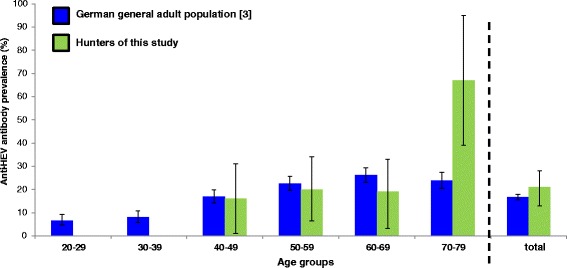
Age-specific anti-HEV prevalence of German general population and the hunters in Wetteraukreis district, Central Germany, 2013. The total and age-specific anti-HEV prevalence of the hunters investigated in this study (green bars) is compared to total and age-specific estimates from a large subsample (*n* = 4352) of sera originating from the 2008–2011 German Health Examination Survey for Adults (Deutscher Erwachsenen Gesundheitssurvey [DEGS]; www.degs-studie.de) representing the baseline prevalence of anti-HEV antibodies in healthy adults in Germany (blue bars) [3]. The 95 % confidence intervals are added per bar as black line

Based on the Pearson’s chi-square test, there was no significant difference between the seroprevalence in the general population in Germany [3] and the hunters of this study (17 *vs.* 21 %, *p* = 0.46). Data from the German Health Examination Survey for Adults were also available for states of Central Germany (17 %, 95 % CI 15–19), which is not significant different from whole Germany (17 %, 95 % CI 16–18). For age-stratified comparisons, we had to use data from whole Germany and were able to confirm that there is no difference between hunters and the German general adult population across most age groups. The only significant difference was observed in the age group of the 70–79 years-olds using Fisher’s exact statistics (24 *vs*. 67 %, *p* < 0.01) (Fig. 2).

### Detection of HEV-specific antibodies and HEV RNA in wild boars from the district

In order to estimate the present or past circulation of HEV in the wild boar population of the district, serum as well as liver and muscle tissue specimens were tested for HEV-specific antibodies and/or HEV RNA. A total of 7 of 46 serum specimens (15 %) from wild boars tested positive for HEV RNA using real-time RT-PCR. After assignment of the specimens to the different hunting areas, the highest HEV incidence was found in wild boars in area SW. For investigation of the distribution of HEV in the boars, available liver and muscle specimens of wild boars were analyzed by real-time RT-PCR (Table 2). All of the wild boars with HEV RNA positive liver specimens had also been tested positive in the blood. The wild boar with the HEV RNA positive muscle specimen had been tested positive in liver and blood. The seroprevalence ranged between 22 and 47 % in the three areas with the highest anti-HEV prevalence detected in the wild boar specimens from the area SW (Table 2).

**Table 2:** HEV RNA and anti-HEV antibody (ab) prevalence in wild boars, Wetteraukreis district, Central Germany, 2013

Hunting area	RNA pos. liver	RNA pos. muscle	RNA pos. serum	Ab pos.
East	1/4 (25 %)	0/4 (0 %)	2/9 (22 %)	2/9 (22 %)
Northwest	0/8 (0 %)	0/8 (0 %)	0/22 (0 %)	10/22 (45 %)
Southwest	3/10 (30 %)	1/10 (10 %)	5/15 (33 %)	7/15 (47 %)
**Total**	**4/22 (18 %)**	**1/22 (4.5 %)**	**7/46 (15 %)**	**19/46 (41 %)**

**Fig. 3:**
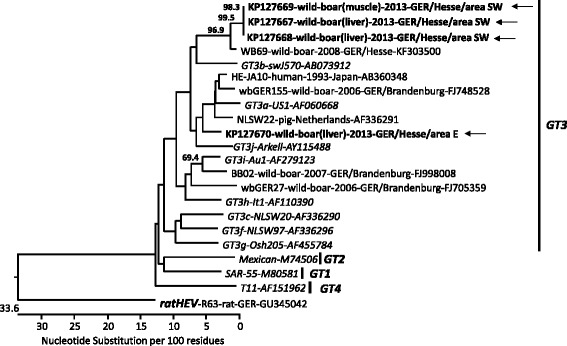
Phylogenetic relationship of HEV sequences derived from wild boars, Wetteraukreis district, Central Germany, 2013. The sequences were selected based on a sequence similarity search using the BLASTn search facility and additional human, pig and wild boar HEV reference strains (in italics) were included; rat HEV was used as an outgroup sequence. The strain designation, source of the viruses (human, pig, wild boar; liver, muscle), year of detection, country/region (GER – Germany), and the GenBank accession numbers are indicated if available. Grouping of the sequences into genotypes is shown on the right. The tree was constructed with a 148 nucleotide fragment of the HEV ORF2 using the neighbour-joining method implemented in the MEGALIGN module of the DNASTAR software package (Lasergene). Bootstrap values >50 % are indicated. HEV sequences derived from wild boars from this study are indicated in boldface and marked with an arrow.

HEV sequences from a 197 nt RT-PCR product were derived from 2 liver and 1 muscle specimens of two wild boars (accession numbers KP127667 to KP127669) of area SW, and from 1 liver specimen of a wild boar (accession number KP127670) from area E. The 148 nt sequences (without primer sequences) from the liver and muscle specimens from area SW were identical to each other and had 88 % nucleotide sequence identity to the sequence from area E. All sequences belonged to gt3, with highest sequence similarities to wild boar strains from Germany, pig strains from the Netherlands and a human strain from Japan as indicated by BLASTn search of the GenBank nr/nt database. A phylogenetic tree set up for the sequences together with closely related strains and reference strains confirmed the different groupings of the strains according to their origin from area SW (subtype 3b) or E (subtype 3a), although the bootstrap support is low due to the short sequence (Fig. 3). Data of the phylogenetic analysis of the hepatitis E virus strains are available at TreeBase with accession number S18364.

### Factors associated with past or present HEV infection of hunters

As all hunters were tested negative for HEV RNA, only factors associated with past HEV infections could be addressed in the analysis. There were only three hunters denying the consumption of any kind of wild boar meat. Nearly all of the hunters (123/126) performed skinning or disembowelling of wild boars at least once per year, the remaining 3 hunters did not answer these questions. About 47 % of the hunters stated to wear protective gloves during skinning or disembowelling wild boars always or nearly always. The proportion of hunters using gloves is highest in the 70–79 year old hunters and lowest in the 30–39 year old hunters (50 vs. 33 %), but without substantial difference among the age groups.

In the univariable analysis, the only factor significantly associated with a positive serology was being older than 70 years (Additional file 1: Table S1). Stratified analysis suggested an association between use of protective gloves and detection of anti-HEV antibodies. Interestingly, this effect was dependent on the respective hunting ground (Additional file 1: Table S2).

In the multivariable model, the interaction between the use of protective gloves and hunting ground was confirmed when adjusted for age. Irrespective of the serological assay used as outcome variable, the highest protective effect of wearing gloves was found for hunting in area SW (Table 3). Based on the result of the Axiom assay, hunters who used protective gloves on a regular basis had an 88 % lower anti-HEV prevalence as compared to hunters disembowelling wild boars in the same area but wearing gloves never, seldom or sometimes (age-adjusted PR 0.12; 95 % CI 0.02–0.86).

**Table 3:** Age-adjusted prevalence ratios by multivariable analysis for hunters, Wetteraukreis district, Hesse, Central Germany, 2013

	Age-adjusted PR (95 % CI)	
	recomWell IgG assay	Axiom assay
Hunting in the area E and use of protective gloves	2.7 (0.47–16)	2.0 (0.98–4.3)
Hunting in the area NW and use of protective gloves	0.27 (0.059–1.3)	0.74 (0.34–1.6)
Hunting in the area SW and use of protective gloves	0.26 (0.033–2.1)	0.12 (0.02–0.86)

## Discussion

This study among hunters in a German rural district aimed to estimate HEV incidence and anti-HEV prevalence in a population being in close contact to HEV animal reservoirs. Our study suggests that the use of protective gloves during skinning and disembowelling of wild boars can prevent exposure to the virus. Protective gloves were regularly used by only about half of the hunters of this study during hunting activities.

Due to the lack of a gold standard [14, 15], anti-HEV prevalence in the hunters was assessed by using two different serological assays. The anti-HEV prevalence determined by using the Axiom assay was nearly double compared to the estimates based on the recomWell IgG assay. Concordance between the two assays used for the human sera was moderate. These discrepancies can be explained by different test principles and antigens.

The hypothesis that hunters are more frequently exposed to HEV and therefore show a higher anti-HEV prevalence than the general adult population could not be confirmed. Seroprevalence data specifically for the general population of Hesse are not available. However, a nti-HEV prevalence in the states of Central Germany was shown not to be different to whole Germany. In the case that this prevalence in Hesse is especially low or high, we might have missed a significant difference between hunters and the general adult population in this region. Due to cumulative lifetime exposure to the virus, anti-HEV prevalence is expected to increase with age. One possible explanation for the abrupt increase of anti-HEV prevalence in the 70–79 year-olds could be a birth cohort effect due to a higher HEV incidence in wild boars in the past or a higher risk of transmission due to more frequent hunting activities or other behavior. Anecdotal reports of hunters in this age group suggest that a more risky behavior without washing hands and no avoidance of contact to blood might also explain this high anti-HEV prevalence in this age group. However, half of them stated that they wear protective gloves always or nearly always during skinning or disemboweling of wild boars. Unfortunately, we do not have information how this behavior might have changed over time. The validity of this finding may be compromised by the small sample size of hunters in this age group (*n* = 12) and needs verification by further studies.

The sampling strategy in this study might have led to a selection bias. On the one hand, it is possible that especially health-conscious hunters were participating in this study not representative in the sense of the use of protective gloves, which may lead to an underestimation of anti-HEV prevalence. On the other hand, if preferential hunters who are worried of an HEV infection because they did not use any gloves were participating, it may have overestimated anti-HEV prevalence.

Our results suggest that factors other than hunting play a major role in the epidemiology of HEV in Germany as for example the consumption of pork meat. Evidence for this transmission route is based on reports from e.g. Japan and France [24, 25]. In large parts of Germany, the consumption of raw pork meat and products is very common. In this study, we did not ask the hunters about the consumption of raw pork meat in the questionnaire and are not able to account for this possible confounder. The consumption of raw pork meat and products may be responsible for a considerable proportion of the detected anti-HEV prevalence in the hunters. To assess the role of pork meat and products as source of HEV infections in Germany, this question should be addressed by a large case-control-study.

The consumption of wild boar meat was not associated with anti-HEV seroprevalence in this study. However, we were able to detect HEV RNA in one of the muscle specimens from a wild boar in SW demonstrating this is a possible transmission route. We have also to mention that the sample size of the hunters was low and nearly all of them consumed wild boar meat, which hampers the identification of consumption of wild boar meat as a factor associated with a past or present HEV infection.

Thus, multivariable analysis focussed on the association between protective measures and serology of the hunters. Irrespective of the serological assay used as outcome variable, the highest effect of wearing gloves was found for hunting in area SW, which was the area with highest HEV incidence and anti-HEV prevalence in the wild boars. The effect of wearing protective gloves when hunting in area E or NW, where wild boars were less tested positive for HEV, seems to be only minor. Since the numbers per stratum are small, confidence intervals are wide and only strong effects can be observed.

The detection of HEV and anti-HEV in the wild boars differed considerably between the three hunting grounds. Consistent with the phylogenetic analysis, these data argue for a localized circulation of the virus within the sounders, small social groups of wild boars consisting of around 20 animals. Thus, HEV prevalence estimations in wild animals may be limited to defined geographical regions and are difficult to predict for other parts of Germany as it is also true for domestic pigs from different regions in Germany [11]. However, the estimated benefit of protective measures during hunting in this study could be affected by participants hunting in more than one of the defined hunting grounds.

These data provide evidence for the recommendation to consider protective measures during hunting [26, 27]. As an additional benefit, wearing gloves can also protect against exposure to other zoonotic pathogens, which can be present in wild boars.

## Conclusions

Hunters may benefit from wearing gloves when in contact with blood or body fluids of HEV animal reservoirs. Thus, this study provides finally scientific evidence for already existing recommendations and should therefore support propagation and tailored communication to persons at risk. However, as the anti-HEV prevalence among these hunters did not significantly differ from that of the general population, other factors may play a more important role in the epidemiology of HEV infection in Germany. This lack of knowledge should be addressed by prospective cohort or large case–control studies in future.

## Additional file

Additional file 1: Table S1. Prevalence ratios by univariable analysis for the hunters, Wetteraukreis district, Hesse in Central Germany, 2013. **Table S2.** Prevalence ratios by stratified analysis for the hunters, Wetteraukreis district, Hesse in Central Germany, 2013. (DOC 84 kb)

## Abbreviations

Ab: Antibody
AR: Attack rate
CI: Confidence interval
DEGS: German Health Examination Survey for Adults (Deutscher Erwachsenen Gesundheitssurvey [DEGS]
E: East
ELISA: Enzyme Linked Immunosorbent Assay
GER: Germany
gt: Genotype
HEV: Hepatitis E virus
IgA: Immunoglobulin A
IgG: Immunoglobulin G
IgM: Immunoglobulin M
κ: Cohen’s kappa coefficient
nt: Nucleotide
NW: Northwest
ORF: Open reading frame
PR: Prevalence ratio
RNA: Ribonucleic acid
RT-PCR: Reverse transcription - polymerase chain reaction
SW: Southwest
