# Supplementary material for: Hepatitis E virus antibody prevalence in hunters from a district in Central Germany, 2013: a cross-sectional study providing evidence for the benefit of protective gloves during disembowelling of wild boars
Source: BMC Infect Dis. 2015 Oct 22;15:440. doi: 10.1186/s12879-015-1199-y (PMC4619084; doi:10.1186/s12879-015-1199-y)
Supplement: Additional file 1: Table S1. — Prevalence ratios by univariable analysis for the hunters, Wetteraukreis district, Hesse in Central Germany, 2013. Table S2. Prevalence ratios by stratified analysis for the hunters, Wetteraukreis district, Hesse in Central Germany, 2013. (DOC 84 kb) [file 12879_2015_1199_MOESM1_ESM.doc]

**Additional file 1**

**Table S1** Prevalence ratios by univariable analysis for the hunters, Wetteraukreis district, Hesse in Central Germany, 2013.

**AR = attack rate; PR = prevalence ratio**

|  |  | | | **Results based on recomWell assay** | | |  |  |  |
| --- | --- | --- | --- | --- | --- | --- | --- | --- | --- |
|  | **AR among exposed** | | | **AR among unexposed** | | | **PR** | **95% CI** | **p-value** |
| **>= 70 years old** | 57% (8/14) | | | 16% (18/112) | | | **3.6** | 1.9-6.6 | 0.002 |
| **Female sex** | 25% (2/8) | | | 20% (24/118) | | | **1.2** | 0.35-4.3 | 0.67 |
| **Hunting in the East** | 26% (9/34) | | | 18% (17/92) | | | **1.4** | 0.71-2.9 | 0.33 |
| **Hunting in the NW** | 14% (9/65) | | | 28% (17/61) | | | **0.50** | 0.24-1.0 | 0.077 |
| **Hunting in the SW** | 30% (8/27) | | | 18% (18/99) | | | **1.6** | 0.80-3.3 | 0.19 |
| **Consumption of wild boar meat** | 21% (26/122) | | | 0% (0/3) | | | **.-.** | .-. | 1.0 |
| **Use of protective gloves always or nearly always** | 14% (8/58) | | | 25% (16/64) | | | **0.55** | 0.26-1.2 | 0.17 |
|  |  |  |  |  |  | **Results based on Axiom assay** |  |  |  |
|  | **AR among exposed** | | | **AR among unexposed** | | | **PR** | **95% CI** | **p-value** |
| **>= 70 years old** | 64% (9/14) | | | 35% (39/111) | | | **1.8** | 1.2-2.9 | 0.044 |
| **Female sex** | 25% (2/8) | | | 39% (46/117) | | | **0.64** | 0.19-2.2 | 0.71 |
| **Hunting in the East** | 52% (17/33) | | | 34% (31/92) | | | **1.5** | 0.99-2.4 | 0.095 |
| **Hunting in the NW** | 31% (20/65) | | | 47% (28/60) | | | **0.66** | 0.42-1.0 | 0.097 |
| **Hunting in the SW** | 41% (11/27) | | | 38% (37/98) | | | **1.1** | 0.64-1.8 | 0.825 |
| **Consumption of wild boar meat** | 39% (47/121) | | | 33% (1/3) | | | **1.2** | 0.23-5.9 | 1.0 |
| **Use of protective gloves always or nearly always** | 37% (21/57) | | | 39% (25/64) | | | **0.94** | 0.60-1.5 | 0.85 |

**Table S2** Prevalence ratios by stratified analysis for the hunters, Wetteraukreis district, Hesse in Central Germany, 2013.

**AR = attack rate; PR = prevalence ratio**

|  | **Results based on recomWell assay** | | | | | |
| --- | --- | --- | --- | --- | --- | --- |
|  |  | **AR among exposed** | **AR among unexposed** | **PR** | **95% CI** | **p-value** |
| **Use of protective gloves always or nearly always** | **Crude** | 14% (8/58) | 25% (16/64) | **0.55** | 0.26-1.2 | 0.17 |
|  | **East** | 22% (2/9) | 22% (5/32) | **1.0** | 0.24-4.3 | 1.0 |
|  | **Northwest** | 6.2% (2/32) | 22% (7/32) | **0.29** | 0.64-1.3 | 0.15 |
|  | **Southwest** | 24% (4/17) | 44% (4/9) | **0.53** | 0.17-1.6 | 0.38 |
|  | **Results based on Axiom assay** | | | | | |
|  |  | **AR among exposed** | **AR among unexposed** | **PR** | **95% CI** | **p-value** |
| **Use of protective gloves always or nearly always** | **Crude** | 37% (21/57) | 39% (25/64) | **0.94** | 0.60-1.5 | 0.85 |
|  | **East** | 75% (6/8) | 39% (9/23) | **1.9** | 1.0-3.6 | 0.11 |
|  | **Northwest** | 34% (11/32) | 28% (9/32) | **1.2** | 0.59-2.5 | 0.79 |
|  | **Southwest** | 24% (4/17) | 78% (7/9) | **0.3** | 0.12-0.76 | 0.014 |
